# Discrimination between the human prostate normal and cancer cell exometabolome by GC-MS

**DOI:** 10.1038/s41598-018-23847-9

**Published:** 2018-04-03

**Authors:** Ana Rita Lima, Ana Margarida Araújo, Joana Pinto, Carmen Jerónimo, Rui Henrique, Maria de Lourdes Bastos, Márcia Carvalho, Paula Guedes de Pinho

**Affiliations:** 1 UCIBIO/REQUIMTE, Department of Biological Sciences, Laboratory of Toxicology, Faculty of Pharmacy, University of Porto, Porto, Portugal; 2 Cancer Biology & Epigenetics Group, Research Center (CI-IPOP) Portuguese Oncology Institute of Porto (IPO Porto), Porto, Portugal; 3 Department of Pathology and Molecular Immunology-Biomedical Sciences Institute (ICBAS), University of Porto, Porto, Portugal; 4 Department of Pathology, Portuguese Oncology Institute of Porto (IPO Porto), Porto, Portugal; 5 UFP Energy, Environment and Health Research Unit (FP-ENAS), University Fernando Pessoa, Porto, Portugal

## Abstract

Serum prostate-specific antigen (PSA) is currently the most used biomarker in clinical practice for prostate cancer (PCa) detection. However, this biomarker has several drawbacks. In this work, an untargeted gas chromatography-mass spectrometry (GC-MS)-based metabolomic profiling of PCa cells was performed to prove the concept that metabolic alterations might differentiate PCa cell lines from normal prostate cell line. For that, we assessed the differences in volatile organic compounds (VOCs) profile in the extracellular medium (exometabolome) of four PCa cell lines and one normal prostate cell line at two pH values (pH 2 and 7) by GC-MS. Multivariate analysis revealed a panel of volatile metabolites that discriminated cancerous from normal prostate cells. The most altered metabolites included ketones, aldehydes and organic acids. Among these, we highlight pentadecane-2-one and decanoic acid, which were significantly increased in PCa compared to normal cells, and cyclohexanone, 4-methylheptan-2-one, 2-methylpentane-1,3-diol, 4-methylbenzaldehyde, 1-(3,5-dimethylfuran-2-yl)ethanone, methyl benzoate and nonanoic acid, which were significantly decreased in PCa cells. The PCa volatilome was markedly influenced by the VOCs extraction pH, though the discriminant capability was similar. Overall, our data suggest that VOCs monitoring has the potential to be used as a PCa screening methodology.

## Introduction

Prostate cancer (PCa) is the second leading cause of cancer-related death in men in most western countries^1^ and for 2017, 26,730 resulting deaths are predicted for USA alone^2^. Prostate-specific antigen (PSA) is currently the most used biomarker for PCa detection, in combination with digital rectal examination (DRE)^3^. However, the role of serum PSA levels as a screening tool for PCa meets with important limitations. Although raised PSA levels (i.e., >4.0 ng/mL) are undoubtedly associated with the presence of PCa, benign conditions, such as prostatitis and benign prostatic hyperplasia (BPH), which are common in the elderly, also cause elevated serum PSA. Consequently, there is a relatively high frequency of unnecessary prostate biopsies, an invasive procedure which is expensive and uncomfortable for the cancer suspects^4^. Moreover, a significant proportion of men with PSA levels within the normal range harbor PCa (up to 22%) and a significant number of these show pathologic features of tumor aggressiveness^5–8^. Indeed, 21% of men with positive end of study biopsies enrolled in the Prostate Cancer Prevention Trial (PCPT) had serum PSA levels between 2.6 and 3.9 ng/mL and 15.4% of the tumors found in men with PSA levels < 2.5 ng/mL were high grade cancers^9^. The PSA test for PCa diagnosis has a area under the curve (AUC) of 0.682 and considering a cut-off of 4.1 ng/ml this biomarker shows a specificity of 93.8% and a sensitivity of 20.5%^1^. Furthermore, PSA is unable to differentiate aggressive from indolent PCa, which may lead to overtreatment^10,11^. Indeed, a large US-based trial found no benefit of PSA screening in reducing PCa-related mortality^12^ and even the large European trial that found a moderate benefit (approx. 20%) acknowledged that 1410 men would have to be screened and additional 48 cases of PCa would need to be treated to avoid a single death from PCa^13^. Besides the human cost, implementing widespread PSA screening for PCa might also lead to double of the total financial costs associated with PCa management^14^. Due to these limitations, the use of PSA for populational screening has been challenged^15^.

Although several molecular tests have been developed over the years and some have already obtained FDA approval^16^, accurate early detection of PCa remains an unmet need. Thus, discovery and validation of novel, more specific and cost-effective biomarkers that might improve early PCa diagnosis and more precisely forecast its clinical behavior in an individual basis is an important research aim.

Metabolomics is a powerful analytical tool in oncology, endowing novel biomarkers and therapeutic targets, as cancer cells have the capacity to modify many homeostatic systems within the body and, consequently, change the production, use and levels of many metabolites^17,18^. A metabolomic approach may allow for the discovery of biochemical signatures, and, consequently, of differences between cancer and healthy metabolic phenotypes^17^ in non-invasive samples. An early intervention is possible using metabolomics, since it is believed that metabolic alterations precede neoplastic proliferation^11^.

Several different matrices may be used in PCa metabolomics studies, but the most common are biofluids (e.g., urine and serum/plasma), tissues and cell lines. Generally, when the chosen matrix is a biofluid and/or a tissue it is very important to be aware that metabolic profile can be altered by factors not related to cancer cell metabolism, like age, diet, drugs, chronobiological variations, among others, which are very important to control to obtain reliable results. On the other hand, experiments performed in cultured cell lines have several advantages compared to the use of urine or plasma, overcoming these problems^17^. In fact, the use of cell lines in preliminary studies have important advantages, as cell lines allow to circumvent several important confounding factors, like age, diet, drugs, chronobiological variations, among others^19–21^. In addition, cell lines have a perfectly defined cell state which allows for the analysis of a target metabolic status. So, we believe that cell lines are the ideal matrix for hypothesis generation and to unveil unchanged metabolic signature originated directly from cells, metabolic alterations that do not appear in studies using animal models or human subjects, due to sample biological complexity, may be revealed^19–21^. Notwithstanding, this *in vitro* model presents some limitations, particularly the fact that cultured cells fail to reproduce the complex cell–cell and cell–matrix interactions in the tumour microenvironment and these interactions are very important for metabolic alterations occurring with tumor progression^20,22^. For these reasons, the findings obtained in *in vitro* studies need further confirmation and validation in biofluids from PCa patients.

Volatilome is defined as the analysis of the volatile profile of a biological system, being accomplished by the evaluation of the volatile organic compounds (VOCs)^23^. VOCs correspond to a carbon-based chemical group, with low molecular weight and high volatility. These compounds are excreted (exometabolome) from the human body in several tissues, accumulated and/or eliminated and can reflect the metabolic condition of an individual^24,25^ and, therefore, have the potential to provide new biomarkers for cancer detection. Since VOCs are the end products of cell metabolism, the volatilome can provide information about intracellular metabolic status. Alterations in VOCs profile may be related to modifications in gene activation, gene expression, proteins and activity of enzymes involved in metabolic pathways^26,27^.

Importantly, the volatilome analysis does not require cell disruption, which allows the use of the same cells at different times^28^. The most commonly used method to analyse VOCs is the Headspace-Solid Phase Microextraction/Gas Chromatography-Mass Spectrometry (HS-SPME/GC-MS), which involves simple and rapid sample preparation, allowing high-throughput screening, has a good sensitivity and does not require a concentration step before analysis^29,30^. Moreover, VOCs can be detected in several different matrices, including exhalated breath and urine, particularly interesting for clinical practice use due to their non-invasive nature^23,31^.

Some reports have already focused on the application of VOCs analysis in different cancers, namely breast^32^, lung^31^, head and neck^33^, esophago-gastric^34^, colorectal skin^35^, skin^36^, liver^37^ and renal^23^, proving to be able to discriminate cancer from normal samples. With great interest to this study, some reports determining VOCs in urine of PCa patients showed significantly differences from urines of control individuals^38,39^.

The main objective of this study is to perform the volatile profiling of four different PCa cell lines (22RV1, PC3; DU145; LNCaP) and one normal prostate epithelial cell line (PNT2) to prove the concept that VOCs emanated into the extracellular medium may discriminate PCa cells from normal prostate cells. VOCs extraction is performed at two different pH values, pH 7 (natural pH of prostate cells’ medium) and pH 2 because it is well established that tumor microenvironment is acidic to promote tumor progression and metastasis^40^. This strategy allows to obtain a more comprehensive evaluation of PCa volatile profile and to infer which pH is optimum for VOCs extraction and detection. VOCs significantly altered as a consequence of PCa metabolism will integrate a panel of candidate biomarkers for early detection of PCa that may, in the future, be translated and validated in other biological matrices (e.g. urine) and used in clinical practice. Furthermore, cell lines included in this study exhibit different hormone-dependency and potential for invasiveness, which allowed for extending our investigation on the metabolites responsible for discriminating PCa cell lines according to tumour androgen-deprivation sensitivity and aggressiveness. To the best of our knowledge, this work is the first to analyze VOCs profile in human PCa cell lines.

## Results

**Figure 1:**
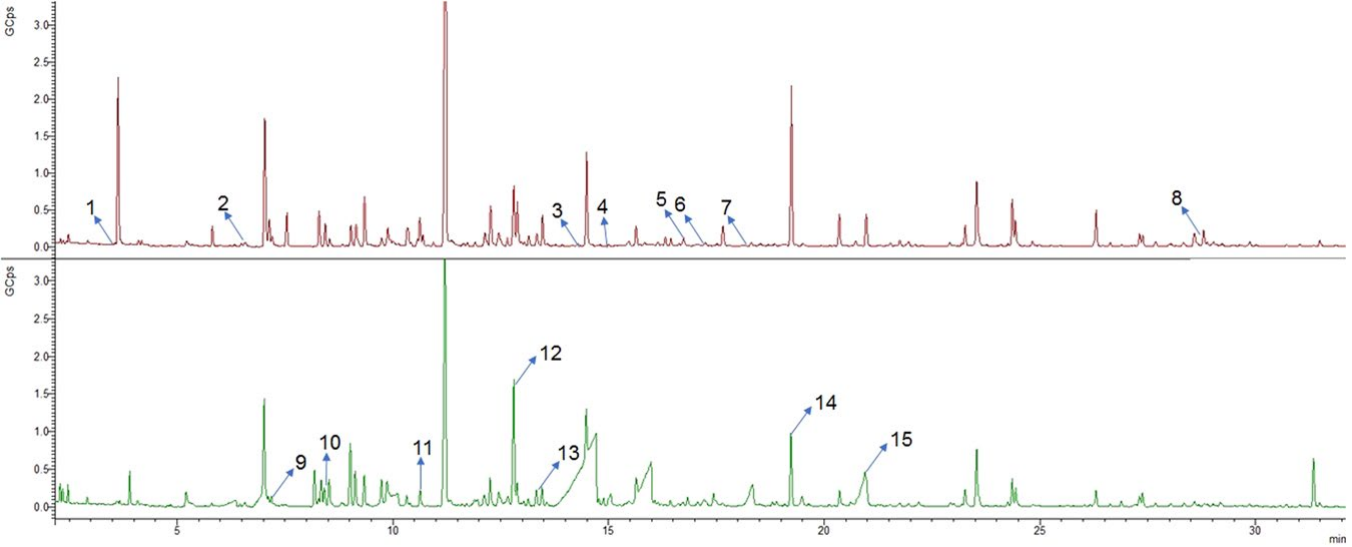
Chromatograms from quality control samples (QCs) at pH 7 and pH 2 (1: 3-methylbut-3-en-2-ol; 2: 1,4-xylene; 3: 2,7-dimethyloctan-1-ol; 4: 2-(4-methylcyclohex-3-en-1-yl)propan2-ol ; 5: 3,7-dimethyloct-7-en-1-ol; 6: 1,3-benzothiazol; 7: decan-1-ol; 8: pentadecan-2-one; 9: cyclohexanone; 10: 4-methyheptan-2-one; 11: 2-methylpentan-1,3-diol; 12: 4-methylbenzaldehyde; 13: methyl benzoate; 14: nonanoic acid; 15: decanoic acid).

In this study, we performed a metabolomic untargeted approach, at two different pHs, pH 7 and pH 2, to evaluate alterations in the volatilome of PCa cell lines (22RV1, PC3, DU145 and LNCaP) when compared with normal prostate cell line (PNT2). A multivariate analysis (MVA) approach was applied to evaluate the ability of volatilome to discriminate the different cell lines used in this study. Figure 1 shows the representative chromatograms of the volatile profile obtained at pH 7 and pH 2. A total of 239 and of 221 features were detected in the chromatograms obtained at pH 7 and pH 2 samples, respectively (Fig. 1).

**Figure 2:**
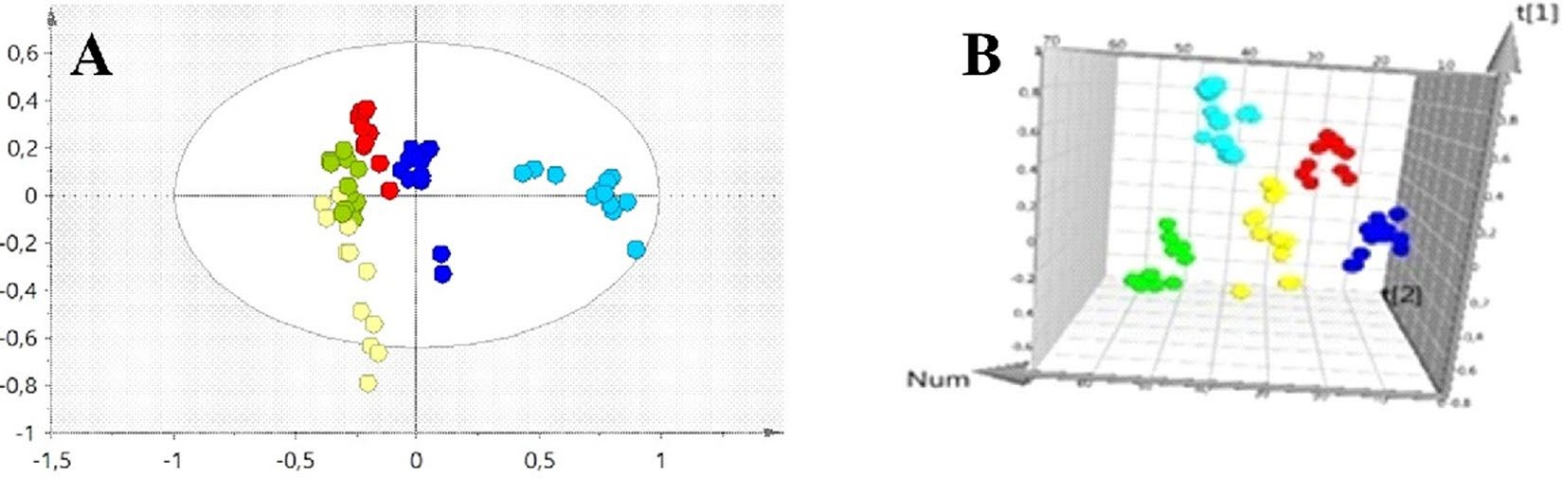
PCA scores scatter plot obtained for the HS-SPME/GC-MS chromatograms of all samples (PNT2: green; 22RV1: dark blue; PC3: light blue; DU145: red; LNCaP: yellow) (**A**) at pH 7 (R^2^X = 0.445) and (**B**) at pH 2 (R^2^X = 0.582). In both PCA it is possible to observe the discriminant capability of the volatilome analyzed by HS-SPME/GC-MS, as each cell line forms an independent cluster.

The reproducibility of the analytical method is evidenced by the QCs cluster in PCA scores plot (Supplementary Fig. 1). Furthermore, the MVA analysis (principal component analysis (PCA) and partial least squares discriminant analysis (PLS-DA)) proved that VOCs can discriminate PCa cell lines from normal prostate cell line and between the different PCa cell lines at both pHs as showed in Fig. 2.

To evaluate which VOCs were responsible for this separation, each cancer cell line was compared separately with the normal cell line, using a MVA supervised analysis (PLS-DA), namely 22RV1 *vs*. PNT2, PC3 *vs*. PNT2, DU145 *vs*. PNT2 *vs*. LNCaP *vs*. PNT2. An optimal separation between PCa cell lines and normal cell line was observed at both pH values (Supplementary Fig. 2 (pH 7) and Supplementary Fig. 3 (pH 2)).

**Figure 3:**
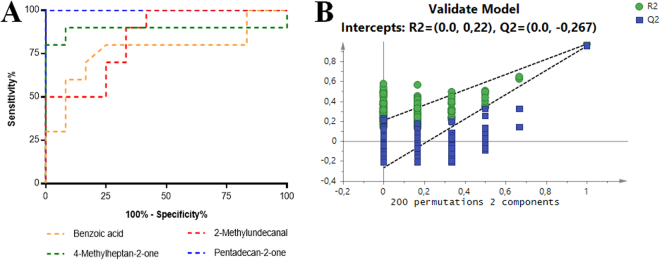
(**A**) Example of assessment of the diagnostic performance through ROC analysis obtained for PC3 *vs* PNT2 at pH7 (2-pentadecanone (AUC = 1) and 2-methylundecanal (AUC = 0.84)) and at pH2 (4-methylheptan-2-one (AUC = 0.89) and benzoic acid (AUC = 0.77). (**B**) Statistical validation of the PLS-DA model for PC3 *vs* PNT2 at pH 2 by permutation testing (200 permutations; 2 components).

To prove the robustness of the discrimination, all PLS-DA models were validated through permutation test (200 random permutations of *Y*-observations, 2 components). The results of this validation showed that all created models were robust for the discrimination between PCa cell lines and the normal prostate cell line (Fig. 3B and Supplementary Table 1).

All VOCs with Variable Importance to the Projection (VIP) values higher than 1 were considered potentially relevant for the separation among cell lines. Hence, at pH 7 a total of 23 VOCs were considered relevant to differentiate 22RV1 from PNT2 and 32 at pH 2; at pH 7, 16 VOCs were considered relevant to differentiate PC3 from PNT2 and 25 at pH 2; at pH 7, 27 VOCs were considered relevant to differentiate DU145 from PNT2 and 32 at pH 2; and, finally, at pH 7, 21 were considered relevant to differentiate LNCaP from PNT2 and 24 at pH 2 (Tables 1 and 2).

**Table 1:** List of VOCS, from the analysis of samples prepared at pH 7, selected as important in discriminating PCa cell lines from normal cell lines (22RV1 *vs* PNT2, PC3 *vs* PNT2, DU145 *vs* PNT2 and LNCaP *vs* PNT2). VOCs are characterized by their IUPAC name. Values for *p*-values percentage of variation, effect size (ES), standard error (ES_SE_) and area under the curve (AUC) are represented for each VOC.

**Chemical name (IUPAC) or common name**	22RV1 *vs* PNT2				PC3 *vs* PNT2				DU145 *vs* PNT2				LNCaP *vs* PNT2			
	p value	Variation ± uncertainty	ES ± ES_SE_	AUC	p value	Variation ± uncertainty	ES ± ES_SE_	AUC	p value	Variation ± uncertainty	ES ± ES_SE_	AUC	p value	Variation ± uncertainty	ES ± ES_SE_	AUC
***Alcohols***																
**3-Methylbut-3-en-2-ol**		↑	↑										**0.0018** ^**P**^	↑**34.78 ± 7.88**	↑**1.48 ± 0.88**	**0.86**
**2,7-Dimethyloctan-1-ol (dihydro-citronellol)**	>0.05	↓	↓		**<0.0001** ^**P**^	↓**74.48 ± 7.90**	↓**5.91 ± 1.84**	**1**	>0.05	↑	↑		>0.05	↓32.61 **±** 28.71	↓	
**2-(4-Methylcyclohex-3-en-1-yl)propan-2-ol (α-Terpineol)**					**<0.0001** ^**P**^	↓**58.47 ±4.51**	↓**7.22 ± 2.18**	**1**								
**3,7-Dimethyloct-7-en-1-ol (α-citronellol)**	**0.0096**	↓**48.80 ± 20.30**	↓**1.26 ± 0.85**	**0.81**	**<0.0001** ^**P**^	↓**58.00 ± 21.18**	↓**1.52 ± 0.88**	**0.98**	**<0.001** ^**P**^	↓**73.13 ± 23.15**	↓**1.86 ± 0.95**	**1**	>0.05	↑137.92 **±** 28.06	↑1.14 **±** 0.84	
**Decan-1-ol**									**0.0251**	↑**18.24 ± 6.65**	↑**1.01 ± 0.84**	**0.77**				
***Esters***																
**1-Methoxypropan-2-yl acetate**	>0.05	↓ 30.40 **±** 16.70	↓0.85 **±** 0.81						>0.05	↑72.03 **±** 8.56	↑1.11 **±** 0.85		**0.0447**	↑**70.57 ± 18.49**	↑ **1.11 ± 0.83**	**0.74**
***Ketones***																
**Pentadecan-2-one**	**<0.0001** ^**P**^	↑ **203.50 ± 5.40**	↑**7.34 ± 2.22**	**1**	**<0.0001** ^**P**^	↑**848.29 ± 6.06**	↑**10.53 ± 3.07**	**1**	**0.0007** ^**P**^	↑**58.66 ± 10.04**	↑**1.89 ± 0.96**	**0.94**	**<0.0001** ^**P**^	↑**73.12 ± 9.26**	↑**2.27 ± 1.01**	**0.98**
***Aromatic hydrocarbon like***																
**1,4-Xylene**					>0.05	↓10.74 **±** 6.87	↓		>0.05	↑89.15 **±** 32.29	↑		**0.0011** ^**P**^	↑**182.61 ± 24.11**	↑**1.56 ± 0.89**	**0.88**
**Naphthalene**	**0.0011** ^**P**^	↑ **49.20 ± 8.80**	↑**1.76 ± 0.92**	**0.88**												
***Aldehydes***																
**2-Methylundecanal**					**0.0029** ^**P**^	↑ **58.67 ± 11.00**	↑**1.54 ± 0.89**	**0.84**	**0.0037**	↓**31.88 ± 10.31**	↓ **1.41 ± 0.88**	**0.84**		↑**71.77 ± 15.15**	↑**1.37 ± 0.86**	**0.82**
***Others***																
**1,3-Benzothiazole**	**<0.0001** ^**P**^	↓**75.00 ± 6.80**	↓**6.91 ± 2.10**	**1**	**<0.0001** ^**P**^	↓**70.22 ± 7.35**	↓**7.03 ± 2.13**	**1**	**0.0348**	↓**23.35 ± 8.56**	↓**1.02 ± 0.84**	**0.70**	**<0.0001** ^**P**^	↓**69.91 ± 7.11**	↓**5.96 ± 1.86**	**1**
**Unknowns**																
**Unknown 2**									**0.0414**	↓**32.26 ± 15.95**	↓**0.92 ± 0.83**	**0.71**				
**Unknown 3**									>0.05	↓20.36 **±** 12.46	↓		**0.0204**	↑**30.56 ± 10.07**	↑**1.04 ± 0.83**	**0.78**
**Unknown 7**	**0.0011** ^**P**^	↓ **58.30 ± 22.20**	↓**1.46 ± 0.88**	**0.88**	>0.05	↓	↓		**0.0010** ^**P**^	↓**23.26 ± 6.27**	↓**1.63 ± 0.91**	**0.85**	**0.0023** ^**P**^	↓**34.10 ± 15.37**	↓**1.05 ± 0.83**	**0.85**
**Unknown 8**	**0.0024**	↑**14.60 ± 3.80**	↑**1.43 ± 0.87**	**0.84**					>0.05	↓9.18 **±** 5.37	↓		>0.05	↑	↑	
**Unknown 12**									**0.0338**	↑**20.39 ± 7.80**	↑**0.96 ± 0.84**	**0.76**				
**Unknown 13**	**<0.0001** ^**P**^	↑**63.50 ± 4.70**	↑**4.03 ± 1.38**	**1**	**<0.0001** ^**P**^	↑**177.78 ± 6.14**	↑**6.03 ± 1.87**	**1**	>0.05	↑	↑		**0.0016** ^**P**^	↑**36.55 ± 7.49**	↑**1.63 ± 0.90**	**0.85**
**Unknown 14**	**0.0003** ^**P**^	↑**58.60 ± 10.10**	↑**1.76 ± 0.92**	**0.91**	**<0.0001** ^**P**^	↑**181.87 ± 4.63**	↑**8.10 ± 2.42**	**1**	>0.05	↓	↓					

^P^Alterations remaining significant after Bonferroni correction, with cutoff *p* value of: 2.17 × 10^−3^ (0.05 divided by 23 analyzed VOCs) for 22RV1 *vs* PNT2; 3.12 × 10^−3^ (0.05 divided by 16 analyzed VOCs) for PC3 *vs* PNT2; 1.85 × 10^−3^ (0.05 divided by 27 analyzed VOCs) for DU145 *vs* PNT2; and 2.38 × 10^−3^ (0.05 divided by 21 analyzed VOCs) for LNCaP *vs* PNT2.

**Table 2:** List of VOCS, from the analysis of samples prepared at pH 2, selected as important in discriminating PCa cell lines from normal cell lines (22RV1 *vs* PNT2, PC3 *vs* PNT2, DU145 *vs* PNT2 and LNCaP *vs* PNT2). VOCs are characterized by their IUPAC name. Values for *p*-values, percentage of variation, effect size (ES), standard error (ES_SE_), and area under the curve (AUC) are represented for each VOC.

**Chemical name (IUPAC) or common name**	22RV1 *vs* PNT2				PC3* vs* PNT2				DU145 *vs* PNT2				LNCaP *vs* PNT2			
	p value	Variation **±** uncertainty	ES **±** ES_SE_	AUC	p value	Variation **±** uncertainty	ES **±** ES_SE_	AUC	p value	Variation **±** uncertainty	ES **±** ES_SE_	AUC	p value	Variation **±** uncertainty	ES **±** ES_SE_	AUC
***Alcohols***																
**2-Methylpentane-1,3-diol**	**0.0105**	↓**10.70 ± 3.80**	↓**1.17 ± 0.84**	**0.81**	**<0.0001** ^**P**^	↓**45.10 ± 5.81**	↓**3.95 ± 1.36**	**1**	**<0.0001** ^**p**^	↓**25.79 ± 3.95**	↓**2.95 ± 1.14**	**0.99**	**<0.0001** ^**p**^	↓**24.26 ± 5.53**	↓**1.97 ± 0.95**	**0.92**
**Phenyl-methanol**	>0.05	↑35.50 **±** 19.60	↑		**0.0189**	↓**33.69 ± 15.30**	↓**1.04 ± 0.83**	**0.82**	>0.05	↑	↑		>0.05	↑	↑	
**2,4-Dimethylheptan-1-ol**	>0.05	↑32.70 **±** 20.10	↑		**0.0210**	↓**35.32 ± 16.50**	↓**1.02 ± 0.83**	**0.79**	>0.05	↓	↓82.41 **±** 7.28		>0.05	↑	↑	
**Phenylethanol**	**<0.0001** ^**P**^	↓**38.10 ± 7.90**	↓**2.36 ± 1.02**	**0.97**	**<0.0001** ^**P**^	↓**69.81 ± 8.12**	↓**5.20 ± 1.66**	**1**	**<0.0001** ^**p**^	↓**38.79 ± 8.76**	↓**2.16 ± 0.99**	**0.92**				
**5-Methyl-2-propan-2-ylcyclohexan-1-ol (DL-menthol)**	>0.05	↓	↓		**0.0068**	↓**45.82 ± 18.48**	↓**1.27 ± 0.85**	**0.83**	**0.0447**	↓**27.4 ± 14.89**	↓**0.84 ± 0.81**	**0.74**	**0.0019** ^**p**^	↓**42.10 ± 13.39**	↓**1.57 ± 0.89**	**0.91**
***Esters***																
**Methyl benzoate**	**<0.0001** ^**P**^	↓**42.20 ± 3.90**	↓**5.51 ± 1.74**	**1**	**<0.0001** ^**P**^	↓**53.12 ± 4.10**	↓**6.95 ± 2.11**	**1**	**<0.0001** ^**p**^	↓**51.06 ± 5.58**	↓**4.84 ± 1.57**	**1**	**<0.0001** ^**P**^	↓**22.45 ± 4.27**	↓**2.33 ± 1.01**	**0.94**
**Benzyl acetate**	**<0.0001** ^**P**^	↓**35.17 ± 6.10**	↓**2.75 ± 1.10**	**1**									**0.0090**	↑**23.73 ± 7.0**	↑**1.19 ± 0.84**	**0.79**
**Methyl nonanoate**	**<0.0001** ^**P**^	↑**366.00 ± 7.90**	↑**6.46 ± 1.98**	**1**												
***Ketones***																
**4-Methylpent-3-en-2-one**	**<0.0001** ^**P**^	↓**47.20 ± 7.10**	↓**3.44 ± 1.24**	**0.99**	**<0.0001** ^**P**^	↓**62.38 ± 7.88**	↓**4.54 ± 1.50**	**1**	**<0.0001** ^**p**^	↓**48.54 ± 9.03**	↓**2.80 ± 1.11**	**0.95**				
**Cyclohexanone**	**<0.0001** ^**P**^	↓**81.10 ± 7.00**	↓**7.72 ± 2.32**	**1**	**<0.0001** ^**P**^	↓**50.48 ± 5.88**	↓**4.53 ± 1.50**	**1**	**<0.0001** ^**p**^	↓**82.41 ± 7.28**	↓**7.59 ± 2.28**	**1**	**<0.0001** ^**P**^	↓**80.15 ± 7.33**	↓**7.19 ± 2.18**	**1**
**4-Methylheptan-2-one**	**<0.0001** ^**P**^	↓**28.60 ± 6.30**	↓**2.10 ± 0.97**	**0.92**	**0.0003** ^**P**^	↓**38.37 ± 8.84**	↓**2.11 ± 0.98**	**0.89**	**<0.0001** ^**p**^	↓**36.06 ± 6.93**	↓**2.51 ± 1.05**	**0.95**	**0.0014** ^**p**^	↓**26.30 ± 7.86**	↓**1.52 ± 0.88**	**0.84**
**5-Methylheptan-2-one**					**<0.0001** ^**P**^	↓**51.58 ± 6.11**	↓**4.48 ± 1.48**	**1**	**<0.0001** ^**p**^	↓**27.47 ± 5.97**	↓**2.10 ± 0.97**	**0.94**	**0.0014** ^**p**^	↓**20.36 ± 5.95**	↓**1.50 ± 0.88**	**0.86**
**1-(3,5-Dimethyl-furan-2-yl) ethanone**	**<0.0001** ^**P**^	↓**47.80 ± 5.30**	↓**4.79 ± 1.56**	**1**	**<0.0001** ^**P**^	↓**41.51 ± 5.09**	↓**4.06 ± 1.38**	**1**	**<0.0001** ^**p**^	↓**46.45 ± 6.01**	↓**3.96 ± 1.36**	**1**	**0.0003** ^**p**^	**21.78 ± 5.50**	↓**1.75 ± 0.91**	**0.88**
**6-Pentyloxan-2-one (δ-decalactone)**	>0.05	↓	↓		**0.0006** ^**P**^	↓**39.93 ± 11.98**	↓**1.640 ± 0.90**	**0.88**	>0.05	↓	↓		>0.05	↑	↑	
***Alkane hydrocarbon***																
**2-Ethoxy-2-methylbutane**	>0.05	↓26.60 **±** 19.90	↓		**0.0342**	↓**38.26 ± 19.77**	↓**0.94 ± 0.81**	**0.74**					>0.05	↓	↓	
**1-Ethoxy-pentane**					0.0056	↑	↑		**0.0010** ^**p**^	↓**31.16 ± 9.21**	↓**1.58 ± 0.89**	**0.88**	**0.0001** ^**p**^	↓**35.34 ± 8.69**	↓**1.95 ± 0.95**	**0.92**
***Aldehydes***																
**4-Methyl-benzaldehyde**	**0.0022**	↓**10.60 ± 3.00**	↓**1.47 ± 0.88**	**0.81**	**<0.0001** ^**P**^	↓**38.00 ± 2.98**	↓**6.20 ± 1.92**	**1**	**<0.0001** ^**p**^	↓**32.04 ± 2.39**	↓**6.29 ± 1.94**	**1**	**0.0005** ^**p**^	↓**18.39 ± 4.31**	↓**1.85 ± 0.93**	**0.87**
**Organic acids**																
**Hexanoic acid**	>0.05	↓	↓		**0.0092**	↓**40.00 ± 16.66**	↓**1.18 ± 0.84**	**0.84**	>0.05	↓	↓		>0.05	↑	↑	
**Benzoic acid**	>0.05	↓	↓		**0.0331**	↓**37.17 ± 17.66**	↓**1.01 ± 0.82**	**0.77**	>0.05	↓	↓		>0.05	↑	↑	
**Nonanoic acid**	**<0.0001** ^**P**^	↓**55.60 ± 8.80**	**3.47 ± 1.25**	**1**	**<0.0001** ^**P**^	**59.85 ± 7.41**	**4.54 ± 1.50**	**1**	**<0.0001** ^**p**^	↑**31.32 ± 6.29**	↑**2.33 ± 1.02**	**0.97**	**<0.0001** ^**P**^	↑**66.28 ± 7.67**	↑**5.09 ± 1.64**	**1**
**4-Methyl-nonanoic acid**	**<0.0001** ^**P**^	↑**1217.10 ± 11.70**	↑**5.79 ± 1.81**	**1**									**<0.0001** ^**P**^	↑**3242.3 ± 10.90**	↑**65.79 ± 2.07**	**1**
**Decanoic acid**	**0.0027**	↑**71.10 ± 14.00**	↑**1.47 ± 0.88**	**0.83**	**<0.0001** ^**P**^	↑**250.44 ± 18.10**	↑**2.42 ± 1.03**	**0.98**	**<0.0001** ^**p**^	↑**268.37 ± 8.66**	↑**5.21 ± 1.67**	**1**	**0.0122**	**74.50 ± 19.00**	↑**1.13 ± 0.83**	**0.80**
**Unknowns**																
**Unknown 16**	>0.05	↓	↓		**0.0321**	↓**30.20 ± 14.74**	↓**0.95 ± 0.82**	**0.79**								
**Unknown 18**	**<0.0001** ^**P**^	↓**94.90 ± 11.80**	↓**6.05 ± 1.88**	**1**	**<0.0001** ^**P**^	↓**59.85 ± 7.41**	↓**4.54 ± 1.50**	**1**								
**Unknown 19**	>0.05	↓20.90 **±** 17.10	↓		**0.0004** ^**P**^	↓**38.38 ± 10.98**	↓**1.71 ± 0.91**	**0.92**	>0.05	↓	↓		>0.05	↑	↑	

↑, VOCs increased; ↓, VOCs decreased in the extracellular medium of PCa compared with normal cell line

^P^Alterations remaining significant after Bonferroni correction, with cutoff p value of 1.56 × 10^−3^ (0.05 divided by 32 analyzed VOCs) for 22RV1 *vs* PNT2; 2.00 × 10^−3^ (0.05 divided by 25 analyzed VOCs) for PC3 *vs* PNT2; 2.38 × 10^−3^ (0.05 divided by 21 analyzed VOCs) for DU145 *vs* PNT2; and 2.08 × 10^−3^ (0.05 divided by 24 analyzed VOCs) for LNCaP *vs* PNT2.

**Figure 4:**
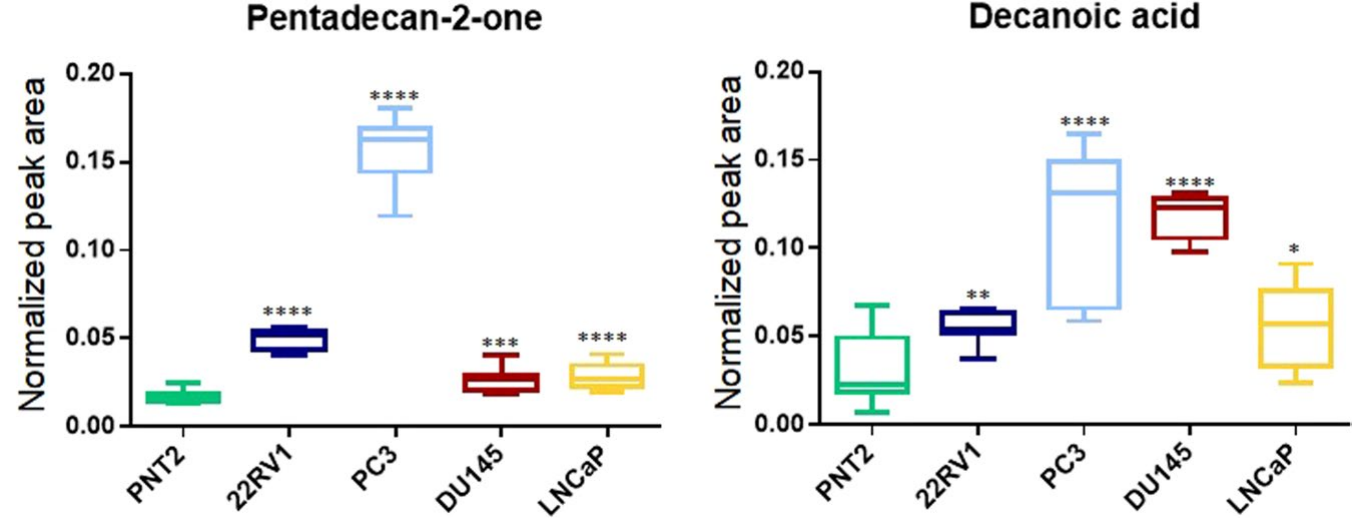
Boxplots from the metabolite pentadecan-2-one, increased in all PCa cells when compared with PNT2 (normal cells), after univariate analysis, obtained at pH 7 and boxplots from the metabolite decanoic acid, increased in all PCa cells when compared with PNT2 (normal cells), after univariate analysis, obtained at pH 2.

To confirm the importance of these metabolites, univariate analysis was performed as explained below (see statistical analysis section). A total of 8 VOCs proved to be relevant to differentiate 22RV1 from PNT2 in the results obtained at pH 7 and 11 at pH 2; 8 VOCs proved to be relevant to differentiate PC3 from PNT2 at pH 7 and 19 at pH 2; 7 VOCs proved to be relevant to differentiate DU145 from PNT2 at pH 7 and 13 at pH 2; and 7 proved to be relevant to differentiate LNCaP from PNT2 at pH7 and 13 at pH 2 (Tables 1 and 2). At pH 7, one VOC stood out, namely, pentadecan-2-one since this VOC revealed to be important for the separation among all PCa cell lines and normal cell line (Fig. 3A, Table 1 and Fig. 4). However, several other VOCs were tentatively identified (Supplementary Table 2) and were also able to discriminate between cancer and normal cell lines, namely, 1,3-benzothiazole, 3,7-dimethyloct-7-en-1-ol (22RV1 *vs* PNT2, PC3 *vs* PNT2, DU145 *vs* PNT2), 2-methylundecanal, (PC3 *vs* PNT2, DU145 *vs* PNT2 and LNCaP *vs* PNT2), 2,7-dimethyloctan-1-ol (PC3 *vs* PNT2), 2-(4-methylcyclohex-3-en-1-yl)propan-2-ol (PC3 *vs* PNT2), decan-1-ol (DU145 *vs* PNT2), 1-methoxypropan-2-yl acetate (LNCaP *vs* PNT2), and 1,4-xylene (LNCaP *vs* PNT2) (Table 1).

**Figure 5:**
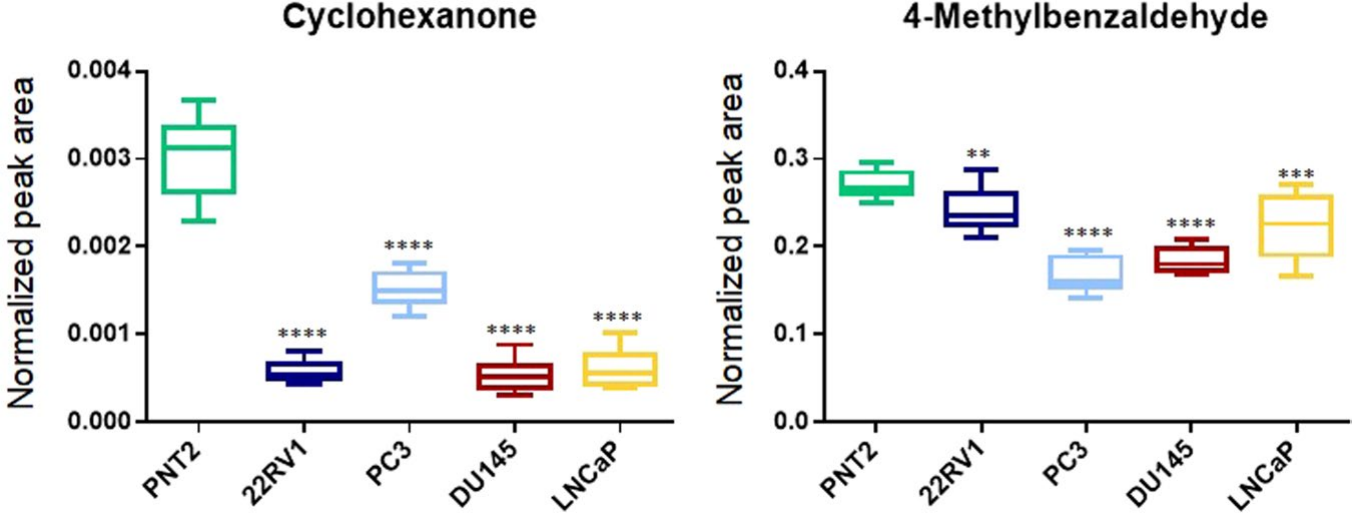
Boxplots from cyclohexanone and 4-methylbenzaldeyde, metabolites decreased in all PCa cells when compared with PNT2 (normal cells), after univariate analysis, obtained at pH 2.

At pH 2, 8 VOCs stood out, namely, cyclohexanone (Fig. 5), 4-methylheptan-2-one, 2-methylpentane-1,3-diol, 4-methylbenzaldehyde (Fig. 5), 1-(3,5-dimethylfuran-2-yl) ethanone, methyl benzoate, nonanoic acid and decanoic acid (Fig. 4) as they revealed to be important for the separation between all PCa cell lines and normal cell line (Table 2). Other VOCs unveiled specificity for each cell line namely, 4-methylpent-3-en-2-one (22RV1 *vs* PNT2, PC3 *vs*. PNT2, DU145 *vs* PNT2), 5-methylheptan-2-one (PC3 *vs* PNT2, DU145 *vs* PNT2 and LNCaP *vs* PNT2), phenylethanol (22RV1 *vs* PNT2, PC3 *vs* PNT2, DU145 *vs* PNT2), 4-methylnonanoic acid (22RV1 *vs* PNT2 and LNCaP *vs* PNT2), benzyl acetate (22RV1 *vs* PNT2 and LNCaP *vs* PNT2), 5-methyl-2-propan-2-ylcyclohexan-1-ol (PC3 *vs* PNT2 and LNCaP *vs* PNT2), 1-ethoxypentane (DU145 *vs* PNT2 and LNCaP *vs* PNT2), methyl nonanoate (22RV1 *vs* PNT2), 2-ethoxy-2-methylbutane (PC3 *vs* PNT2), hexanoic acid (PC3 *vs* PNT2), phenylmethanol (PC3 *vs* PNT2), 2,4-dimethylheptan-1-ol (PC3 *vs*. PNT2), benzoic acid (PC3 *vs* PNT2), 6-pentyloxan-2-one (PC3 *vs* PNT2) (Table 2). Some unidentified VOCs (codified as unknown 1, 2, 3…) were also found as important for the discrimination between cancer and normal cell lines.

ROC curves were performed for all significantly altered VOCs (Fig. 3A), and the results revealed that all significantly altered metabolites have a AUC higher than 0.5 and several metabolites showed a AUC equal to 1 (Tables 1 and 2).

The sensitivity and specificity of the discriminative sets of metabolites obtained for each pair were also calculated and the results reveal that the discriminant sets have a sensitivity and specificity of 100% or very close to this value for discriminating the PCa cell lines from the normal prostate cell line (Supplementary Table 3).

## Discussion

### Comparative analysis of VOCs in PCa and prostate normal cell lines obtained at pH 7

In this work, we showed the potential of VOCs at physiological pH to discriminate several PCa cell lines from a normal prostate cell line. The results disclosed significantly altered VOCs in all PCa cell lines when compared with the normal cell line (Table 1), with special emphasis for pentadecan-2-one that was found increased in all PCa cell lines. Importantly, this VOC seems to be cell-specific as it is not present in the culture medium, and, therefore, it should be investigated in future as a promising biomarker for PCa.

In addition, our findings further demonstrate that it is possible to discriminate PCa cells with different degrees of aggressiveness based on VOCs profiling. For instance, 2,7-dimethyloctan-1-ol and 2-(4-methylcyclohex-3-en-1-yl)propan-2-ol were found significantly decreased in the culture medium of PC3 cells, which have high metastatic potential (Table 1). The significant increase of decan-1-ol levels was a characteristic alteration of the cell line with moderate metastatic potential (DU145), whereas the significant increase of 3-methylbut-3-en-2-ol, 1-methoxypropan-2-yl acetate and 1,4-xylene levels were characteristic of the cell line with the lower metastatic potential (LNCaP) (Table 1). It was also possible to discriminate androgen responsive from androgen non-responsive cell lines using 3,7-dimethyloct-7-en-1-ol, since this metabolite was only significantly decreased in androgen non-responsive cell lines (PC3, DU145 and 22RV1) (Table 1). Interestingly, 22RV1 that is an androgen receptor positive cell line was able to survive and grow in a culture medium not supplemented with androgen, resulting in the development of an androgen-independent phenotype cell line, possibly by a mechanism similar to the one occurring in clinical practice after hormone deprivation therapy. This explanation was already addressed by other researchers^41^.

The integration of VOCs in metabolic pathways is, at present, hard to perform, because studies with volatilome are scarce and very recent. However, using the human metabolome database (HMDB)^42–44^, it was possible to see if some of those metabolites found in our samples were already found by other researchers in biological matrices. The Supplementary Table 2 enables to do the interconnection between the significantly altered identified metabolites in our *in vitro* disease model and this database^42–44^.

The increase in pentadecan-2-one observed in all PCa cell lines is in accordance with previously reported increase in ketone levels in urine from PCa patients^38^, as well as in other cancer cell lines^37,39,45,46^. This finding may be explained by the increased fatty acid β-oxidation and protein metabolism, used to produce energy, which lead to ketones production^47^. In particular, the metabolism of C15:0 long-chain fatty acids may originate pentadecan-2-one in PCa cell lines^48^. Furthermore, ketones can also be produced from the respective secondary alcohols^37^. Alteration in the activity of alcohol dehydrogenase^49^ and cytochrome P450 (CYP 450) activities^30^ can also be related to alteration in ketone and aldehydes levels, since hydrocarbons can be metabolized to aldehydes or ketones in the human body via these enzymes^50^. In our study, alterations in the aldehydes and hydrocarbons levels were also observed, which reinforce the connection between the observed changes and alterations in the activity of these two enzymes.

The alteration in aldehydes (e.g. 2-methylundecanal) profiling is common in urine of PCa patients^38^ and in cancer cell lines from colon and lung^37,39,51^. This altered profile may be associated with an alteration in lipid peroxidation, induced by the increased ROS levels, characteristic of cancer cell metabolism and inflammation^38,47,51,52^. Beyond lipid peroxidation, aldehydes can also result from amino acid and carbohydrate catabolism^53,54^. An alternative explanation for the aldehyde levels variation can be related to alterations in aldehyde dehydrogenases (ALDHs) activity, responsible for the aldehydes oxidation to carboxylic acids^53,54^. Furthermore, ALDHs are involved in cellular proliferation, differentiation and survival, and in the cellular response to oxidative stress. The alteration in ALDHs was associated with PCa and other cancers progression^53,54^.

A significant alteration in aromatic hydrocarbons, such as naphthalene and 1,4-xylene, in the culture media of cancer cell lines was also observed. Importantly, the alteration in 1,4-xylene levels observed in our study is in concordance with previously reported alteration in 1,4-xylene in exhaled breath from PCa patients^55^ and in urines from head and neck cancer patients^56^. The presence of these molecules was previously related with the presence of ROS and oxidative stress^47,57,58^. So, the increase in aromatic hydrocarbons in cancer cell lines media can be an indicator of an increase in ROS activity, which could lead to lipid peroxidation and consequently to cell membrane damage^59^.

In our study, several alcohols, such as 3-methylbut-3-en-2-ol, 2,7-dimethyloctan-1-ol, 2-(4-methylcyclohex-3-en-1-yl)propan-2-ol, 3,7-dimethyloct-7-en-1-ol and decan-1-ol were altered in PCa cell lines; this alteration in alcohols levels may be due to the alteration in hydrocarbon metabolism, being alcohols end-products of this metabolism^50,58^. The β-oxidation used by cancer cells to produce acetyl-CoA, that can be incorporated in Krebs cycle, may be other important source of alcohols. Thus, one may hypothesize that decan-1-ol can be associated with the synthesis of a medium-chain fatty acid, namely capric acid (C10:0), for energy production^42–44^. Other well described characteristic of cancer cells is their rapid growth, which implies an increase of cellular membrane synthesis. The alcohols may be metabolized to carboxylic acids and these acids further used to the synthesis of cellular membrane precursors^60^. Another possible explanation for the increased levels of some alcohols is the induction of the cytochrome P450 enzymes that occurs during carcinogenesis^56^. CYP 450 can hydroxylate several VOCs, including the alkanes produced during lipid peroxidation of polyunsaturated fatty acids, which leads to the production of corresponding alcohols^56^. Alcohols can also be produced by enzymatic reduction of aldehydes by alcohol dehydrogenases^37^.

### Comparative analysis of VOCs in PCa and normal cell lines obtained at pH 2

It is well established that tumor microenvironment is acidic^40^, to promote tumor progression and metastasis. The biochemical mechanisms related with acidic pH include hypoxia, excessive glycolysis, hyperexpression of carbonic anhydrase and poor perfusion^40^. The important role of acidic pH in tumor microenvironment highlights the interest of studying the volatilome at pH 2.

As for physiological pH, the volatilome obtained at pH 2 was able to differentiate PCa cell lines from normal prostate cell line, taking into account the quality of the two PLS-DA models (R^2^X = 0.526; R^2^Y = 0.457; Q^2^ = 0.437 for pH 2 *vs* R^2^X = 0.445; R^2^Y = 0.463; Q^2^ = 0.446 for pH 7). Although the discriminant capability of these two models is similar, the discriminant metabolites were not the same at the two medium pH, showing that acidification of the samples markedly influences the prostate cell volatilome (e.g. detection of volatile organic acids). As described by other authors^30^, the detected volatilome was markedly different in the two pHs in study, which highlights the importance of the study of volatilome at different pH, to obtain a comprehensive picture of cells volatilome.

Seven VOCs, namely 2-methylpentane-1,3-diol, 1-(3,5-dimethylfuran-2-yl)ethanone, methyl benzoate, nonanoic acid, cyclohexanone, 4-methylbenzaldehyde (Fig. 5), and 4-methylheptan-2-one were significantly decreased in the extracellular medium of all PCa cell lines when compared with normal cell line (Table 2). On the other hand, decanoic acid (Fig. 4) was increased in the extracellular medium of all PCa cell lines. Based on the altered volatile profile among cell lines in study, it was also possible to discriminate PCa cells with different aggressiveness. For example, 2-ethoxy-2-methylbutane acid, hexanoic acid, phenylmethanol, 2,4-dimethylheptan-1-ol, benzoic acid, and 6-pentyloxan-2-one were significantly decreased in the extracellular medium of high metastatic potential cell line (PC3), whereas 1-ethoxypentane was significantly decreased specifically in moderate and low metastatic potential cell line (DU145 and LNCaP, respectively). Moreover, some VOCs can discriminate androgen responsive cell lines from androgen non-responsive cell lines. For instance, 4-methylpent-3-en-2-one and phenylethanol (Table 2) were only significantly decreased in the androgen non-responsive cell lines (PC3, DU145 and 22RV1). Importantly, 4-methylnonanoic acid, hexanoic acid and 6-pentyloxan-2-one were absent in the cellular medium, suggesting its origin from cellular metabolism, thus unveiling great potential as candidate biomarkers to be further validated in biofluids from PCa patients.

The majority of the VOCs significantly altered were decreased in cancer cell lines in comparison with normal cell line, being this observation previously made in other metabolomic studies with urine from PCa patients^38,61^. The cancer cells may use these metabolites for their metabolic processes more extensively then normal prostate cells^38,61^, converting these VOCs in other metabolites that cannot be detected by our methodology.

Comparison of the metabolites found in our samples at pH 2 was made with those already described by other researchers in biological matrices^42–44^ (Supplementary Table 2). The significant alterations in the levels of decanoic acid in the PCa cells study is in concordance with previously reported in serum of PCa patients^62^. Also, alterations in decanoic acid, phenylmethanol and nonanoic acid were associated with melanoma^63^. The significant alteration in the levels of 4-methylheptan-2-one study is in concordance with previously reported in urines from head and neck cancer patients^56^.

Similarly to pH 7, in the volatile profile obtained at pH 2, it was observed a significant alteration in the levels of ketones, aldehydes and alcohols associated with PCa. Significant alterations in levels of some esters and organic acids were also observed.

Esters can be found naturally in fats and may be originated from acids and alcohols during lipid hydrolysis, which may explain the reduction in the levels of these VOCs, observed in PCa cell lines^50^. So, the alteration in esters levels and organic acids levels can be related as ester metabolism can lead to organic acid production and acid metabolism can lead esters formation^37^.

Alteration in organic acids was previously described in urine and serum of PCa patients^61,62^. Organic acids can be involved in several biological processes, including cell signaling, energy storage, energy source, and cellular membrane integrity^61^. As aforementioned, alteration in ALDHs function can also be associated with the alteration in the organic acid levels, as this enzyme can catalyze the conversion of aldehydes into organic acids^37^.

## Conclusion

Our results highlight the capability of volatilome analysis to identify potential biomarkers to be used in PCa diagnosis, since at both analyzed pH the VOCs profile was able of differentiate all cell lines in study. One of the most problematic drawbacks of PSA is its inability to differentiate aggressive PCa from indolent PCa. Our results reveal that through the evaluation of the alterations in volatile profile of PCa cell lines it is possible to differentiate PCa cells with different aggressiveness. The use in clinical practice of a biomarker or a panel of biomarkers able to make this differentiation will bring major benefits for PCa patients, improving PCa management and circumventing the problem of overtreatment. Furthermore, volatilome also prove be able to differentiate PCa cell lines with different hormonal dependency.

The influence of pH in the volatilome was also evaluated and is a parameter to be considered. Acidification of the samples markedly influenced the volatile composition (e.g. detection of volatile organic acids), hence, to have a more comprehensive analysis of cell volatilome it is advantageous to use both pH.

The integration of VOCs in specific metabolic pathways is still very difficult, needing more studies to completely accomplish this goal. Nevertheless, our results indicate that PCa cell lines have lipid metabolism altered, mainly in lipid peroxidation, lipid hydrolysis, synthesis of fatty acid and fatty acid β-oxidation. The obtained results also suggest the modification in the activity of some important enzymes, namely, alcohol dehydrogenase, cytochrome P450 and aldehyde dehydrogenase.

It is important to emphasize that for a possible use in the clinical practice it is mandatory to prove the translatability of these results to *in vivo* samples, preferentially urine. Further studies are still needed to confirm our results, and find out a non-invasive, sensible and specific biomarker (or set of biomarkers) for prostate cancer.

## Materials and Methods

### Chemicals

All chemicals and reagents were of analytical grade. RPMI-1640 medium, 4-fluorobenzaldeyde (internal standard) and all GC-MS standard compounds were purchased from Sigma-Aldrich Co. (St. Louis, MO, USA). The antibiotic mixture penicillin/streptomycin (10000 U/mL/10000 µg/mL), heat inactivated fetal bovine serum (FBS) and trypsin 0.25%-EDTA were purchased from GIBCO Invitrogen (Barcelona, Spain). Hydrochloric acid (HCl) and sodium hydrogen carbonate were obtained from Merck (Darmstadt, Germany). Sodium chloride (NaCl) was from VWR (Leuven, Belgium).

### Cell Culture

PCa human immortalized cell lines (PC3, 22RV1, DU145 and LNCaP) and human normal prostate epithelium immortalized cell line (PNT2) were provided by Portuguese Oncology Institute-Porto (IPO-Porto) and their respective characteristics are shown in Supplementary Table 4. In brief, 22RV1 and LNCaP are cell lines from prostate carcinoma with androgen receptor (AR) expression, whereas PC3 and DU145 cells lines are androgen unresponsive. PC3 is a cell line from grade IV adenocarcinoma with high metastatic potential; DU145 is a cell line from prostate carcinoma, with moderated metastatic potential; and LNCaP is a cell line from prostate carcinoma with low metastatic potential. The selection of the cells lines was performed to allow to cover an extended range of PCa with different features, namely different aggressiveness and hormonal dependency. Furthermore, it was possible to use the same culture media to grow all cell lines. The use of different culture media, in this kind of studies, may be a confounding factor, once different culture media might have different effects on cellular metabolic profiles, thereby compromising the comparison of the VOCs profiles^64^.

All cell lines were grown in RPMI-1640 supplemented with 10% of FBS and 1% of penicillin/streptomycin, maintained at 37 °C and 5% CO_2_ in 75 cm^2^ culture flasks and grown to 80% confluency, before each passage. For all cell lines the same passages were used. All cell lines were routinely tested for Mycoplasma spp. contamination (PCR Mycoplasma Detection Set, Clontech Laboratories).

### VOCs collection and extraction

The experiments were carried out during 4 consecutive passages (passages 3 to 6) with triplicates for each passage, after an adaptation stage of at least 3 passages for all cell cultures. After the cells had reached near confluence, the medium was discarded and replaced with 15 mL of fresh RMPI-1640 medium, and incubated at 37 °C and 5% CO_2_ for 48 h, together with three blanks (cellular medium without cells treated exactly as the study samples). After 48 h, the extracellular medium from flasks with cells and blanks were collected, centrifuged (2000 × g for 10 min at 4 °C), the supernatant separated from the pellet, and divided into two equal aliquots (one for analysis at pH 7 and another for analysis at pH 2). To prevent the loss of VOCs, all collection steps were performed on ice and immediately frozen at −80 °C until analysis.

Stored samples were thawed slowly at low temperatures, in order to minimize the loss of VOCs^65^. All samples were analyzed at the pH of the medium culture around pH 7 (pH = 7.675 ± 0.28) and at pH 2 (pH = 2.156 ± 0.31). For acidification, a fixed volume of 5 M HCl was added. For GC-MS analysis, 2 mL of sample were placed into a 10 mL glass vial with the internal standard (4-fluorobenzaldehyde (10 µg/mL)) and NaCl (≈0.59 g). The extraction was performed according to the conditions previously determined^65^. Simultaneously, quality control (QCs) samples were also prepared using the same protocol. QCs samples were a pool of all samples and blanks used in the study. To avoid the frequent freezing and thawing, that could change the VOCs fingerprint, the QCs samples were divided in several aliquots and each aliquot was defrost prior to GC-MS analysis.

### GC–MS analysis

The data acquisition was performed in a Scion-436 gas chromatograph coupled to a Bruker SQ (single quadrupole) equipped with a SCION SQ ion trap mass detector and a Bruker Daltonics MS workstation software version 6.8, with a Rxi-5Sil MS (30 m × 0.25 mm × 0.25 µm) column from RESTEK. A CombiPAL automatic autosampler (Varian, Palo Alto, CA) was used and experimental conditions were previously described^65^. The carrier gas used was helium C-60 (Gasin, Portugal) (flow of 1 mL/min) and the injector port was heated to 230 °C. The analysis was performed in Full Scan mode. The oven temperature was fixed at 40 °C for 1 min, then increasing to 250 °C (rate 5 °C/min), held for 5 min, then increasing to 300 °C (rate 5 °C/min) and held for 1 min. The transfer line temperature was 280 °C, manifold temperature was 50 °C and the trap temperature was 180 °C. The mass range was 40–350 m/z, with a scan rate of 6 scan/s. All samples were injected randomly^65^.

To ensure reproducibility, QCs samples were injected at the same conditions on every 6 samples (three times *per* day)^66^.

The identification of VOCs was accomplished by using standards, the National Institute of Standards and Technology (NIST 14) data base spectra library and by comparing experimental Kovats index and Kovats index from literature (Supplementary Table 2).

### Statistical analysis

Prior to statistical analysis of results, all chromatograms were pre-processed. This pre-processing includes: baseline correction, peak detection, chromatogram deconvolution and alignment, performed using the program MZmine^67^. The parameters used to accomplish these were: RT range 2.8–34.0 min; m/z range 50–250; MS data noise level 1.0 × 10^4^; m/z tolerance 0.5; chromatogram baseline level 1.0 × 10^3^; peak duration range 0.02–0.30 min. Also, all ions with a relative standard deviation^68^ greater than 30% as well as ions (m/z) coming from the column, the fiber, among others were manually removed from the matrix. The obtained data was normalized for the total area of the chromatograms.

The statistical treatment includes unsupervised (PCA) and supervised analyzes (PLS-DA). All VOCs with VIP values greater than one were considered potential relevant for the separation among cell lines. For these relevant compounds, univariate analysis was performed, using Shapiro-Wilk test, to determine normality distribution of data, and unpaired Student’s t-test with Welch correction test, for normal distribution, or unpaired Mann-Whitney test, for non-normal distribution, to calculate the *p* value. In addition, the percentage of variation, uncertainty of the percentage of variation, and effect size and the standard error were calculated^69^. Bonferroni correction was used to adjust *p*-values. For all significantly (*p* value < 0.05, effect size higher than the standard error and % variation superior to uncertainty) altered metabolites, receiver operating characteristic^10^ curves were computed and AUC (area under the curve) was also calculated (using MetaboAnalyst 3.0).

Finally, to confirm the robustness of the PLS-DA models, all PLS-DA models were validated through permutation test (200 random permutations of *Y*-observations, 2 components). The sensitivity and specificity of the discriminant sets of metabolites obtained from all comparison pairs at pH 7 and pH 2 were also calculated (using MetaboAnalyst 3.0).

## Electronic supplementary material

Supplemetary figures and tables
