# Supplementary material for: Discrimination between the human prostate normal and cancer cell exometabolome by GC-MS
Source: Sci Rep. 2018 Apr 3;8:5539. doi: 10.1038/s41598-018-23847-9 (PMC5882858; doi:10.1038/s41598-018-23847-9)
Supplement: Supplementary file 1 — Supplemetary figures and tables [file 41598_2018_23847_MOESM1_ESM.pdf]

Discrimination between the human prostate normal and cancer cell exometabolome by GC-MS

Ana Rita Lima[1], Ana Margarida Araújo[1], Joana Pinto[1], Carmen Jerónimo[2,3], Rui Henrique[2,3,4], Maria de Lourdes Bastos[1], Márcia Carvalho[1,5], Paula Guedes de Pinho[1]

[1]UCIBIO/REQUIMTE, Department of Biological Sciences, Laboratory of Toxicology, Faculty of Pharmacy, University of Porto, Porto, Portugal

[2]Cancer Biology & Epigenetics Group, Research Center (CI-IPOP) Portuguese Oncology Institute of Porto (IPO Porto), Porto, Portugal.

[3]Department of Pathology and Molecular Immunology-Biomedical Sciences Institute (ICBAS), University of Porto, Porto, Portugal.

[4]Department of Pathology, Portuguese Oncology Institute of Porto (IPO Porto), Porto, Portugal.

[5]UFP Energy, Environment and Health Research Unit (FP-ENAS), University Fernando Pessoa, Porto, Portugal.

***Corresponding authors:**

E-mail addresses:

ritacmlima@hotmail.com (A.R.L.)

pguedes@ff.up.pt (P.G.P)

Address:

REQUIMTE, Laboratory of Toxicology

Department of Biological Sciences

Faculty of Pharmacy

University of Porto

Rua Jorge Viterbo Ferreira, 228

4050-313 Porto, Portugal

Tel.: +351 220428599; fax: +351 226093390

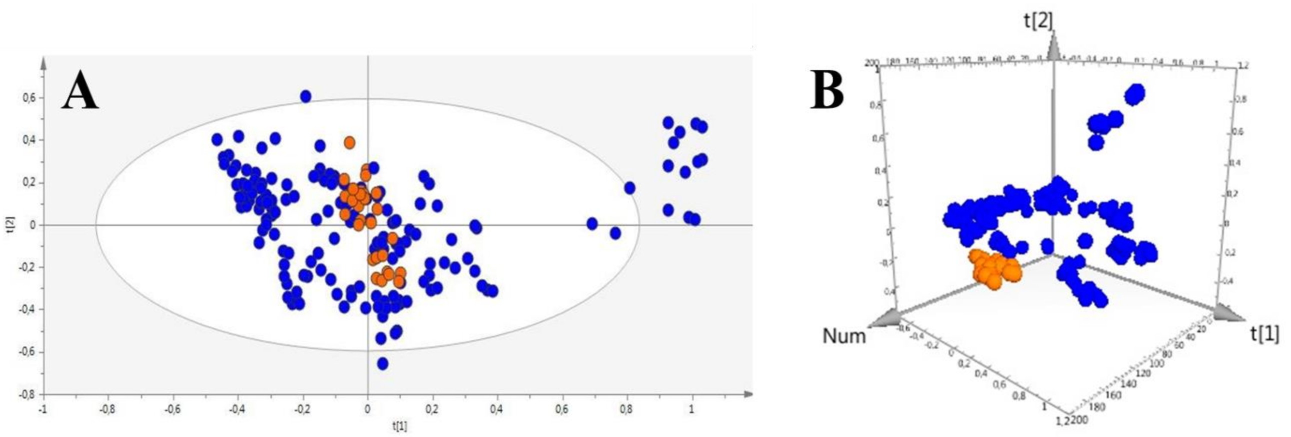

Figure 1: PCA scores scatter plot obtained for the HS-SPME/GC-MS chromatograms of all samples, namely QCs samples (in orange), and the extracellular medium of all cell lines and blanks (blue) (**A**) at pH 7 ($R^2X = 0.357$) and (**B**) at pH 2 ($R^2X = 0.562$). In both PCA, it is possible to observe a cluster with all QCs which proves the reproducibility of the analytical approach.

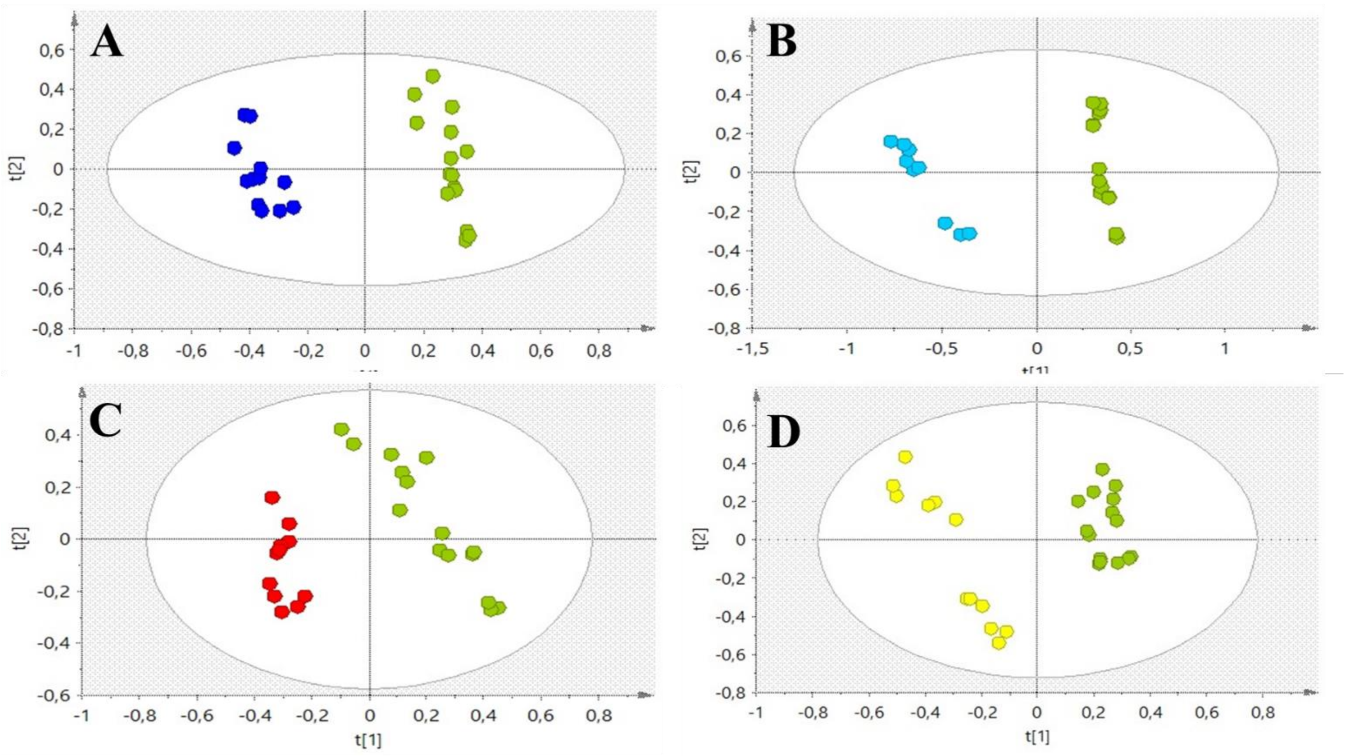

Figure 2: PLS-DA obtained from PCa cell line vs normal cell line at pH 7. (**A):** 22RV1 (dark blue) vs PNT2 (green) (R^2X=0.436; R^2Y=0.989; Q^2=0.967). (**B):** (light blue) vs PNT2 (green) (R^2X=0.667; R^2Y=0.985; Q^2=0.973). (**C):** DU145 (red) vs PNT2 (green) (R^2X=0.401; R^2Y=0.959; Q^2=0.884). (**D):** LNCaP (yellow) vs PNT2 (green) (R^2X=0.486; R^2Y=0.97; Q^2=0.931). In all PLS-DA, it is possible to observe the discriminant capability of the exometabolome analyzed by HS-SPME/GC-MS to differentiate PCa cell lines from the normal cell line, as each cell line forms an independent cluster Q^2>0.5.

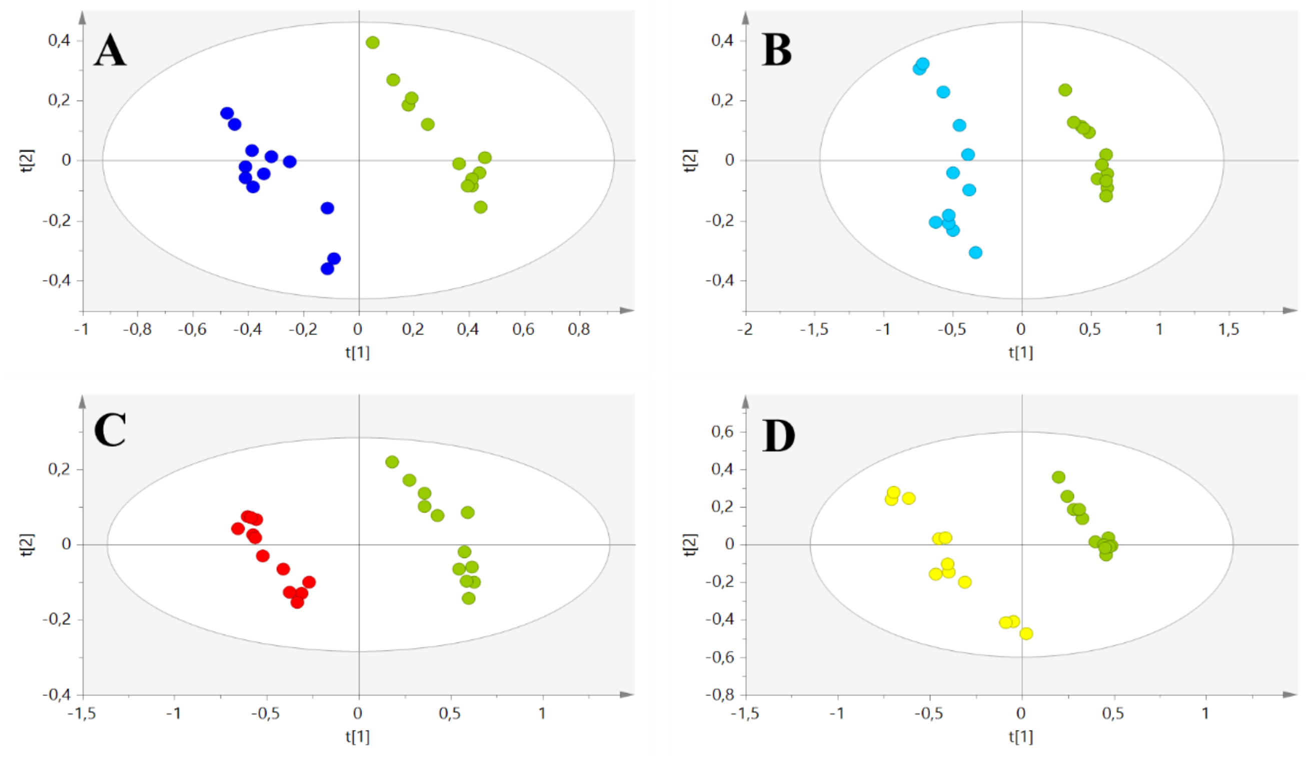

Figure 3: PLS-DA obtained from PCa vs PNT2 at pH 2. **A.** 22RV1 (dark blue) vs PNT2 (green) (R^2X=0.586; R^2Y=0.976; Q^2=0.956). **B.** PC3 (light blue) vs PNT2 (green) (R^2X=0.721; R^2Y=0.976; Q^2=0.957). **C.** DU145 (red) vs PNT2 (green) (R^2X=0.788; R^2Y=0.987; Q^2=0.969). **D.** LNCaP (yellow) vs PNT2 (green) (R^2X=0.729; R^2Y=0.989; Q^2=0.983). In all PLS-DA it is possible to observe the discriminant capability of the exometabolome analyzed by HS-SPME/GC-MS to differentiate PCa cell lines from the normal cell line, as each cell line forms an independent cluster and Q^2>0.5.

Table 1: Statistical validation of the PLS-DA models, obtained for pH 7 and pH 2, by permutation testing (200 permutations)

**Models**	**Interception**	
	**Q^2**	**R^2**
**pH7**		
22RV1 vs PNT2	(0.0; -0.37)	(0.0; -0.377)
PC3 vs PNT2	(0.0; -0.275)	(0.0; -0.301)
DU145 vs PNT2	(0.0; -0.321)	(0.0; -0.447)
LNCaP vs PNT2	(0.0; -0.352)	0.402
**pH2**		
22RV1 vs PNT2	(0.0; -0.163)	(0.0; -0.442)
PC3 vs PNT2	(0.0; -0.267)	(0.0; -0.22)
DU145 vs PNT2	(0.0; -0.0372)	(0.0; -0.434)
LNCaP vs PNT2	(0.0; -0.187)	(0.0; -0.323)

Table 2: List of VOCs selected from PLS-DA of 22RV1 vs PNT2, PC3 vs PNT2, DU145 vs PNT2 and LNCaP vs PNT2 (VIP>1) as potentially important for discrimination between PCa and normal cell lines obtained at pH 7 and pH 2. The identification of the metabolites is based on the NIST (2014) and standards. They are characterized by their IUPAC name, RT, characteristic ions, Kovat indices from literature, experimental Kovat indices, NIST R-match, Cas registry number, HMDB code (when available), as well as cellular locations.

Chemical name (IUPAC) or common name	RT	Characteristic ions	KI from literature	Experimental KI	MS-R match	Cas number	HMDB number	Cellular locations
**2-Ethoxy-2-methylbutane ***	2.49	59	728	707	683	919-94-8	NA	NA
**3-Methylbut-3-en-2-ol** γ	2.95	58	737	725	724	10473-14-0	HMDB39779	Cytoplasm<br>Extracellular
**3-Methylbut-3-en-1-ol** γ	3.57	67/68	730	749	881	763-32-6	HMDB30126	Cytoplasm<br>Extracellular<br>Membrane
**1-Ethoxypentane ***	3.63	59/70	760	752	786	17952-11-3	NA	NA
**4-Methylpentan-2-one *** γ	3.71	58	735	755	907	108-10-1	NA	NA
**Unknown 15 ***	4.18	70	NA	773	NA	NA	NA	NA
**4-Methylpent-3-en-2-one*** γ	4.81	55/83/98	798	798	855	141-79-7	HMDB31563	Cytoplasm<br>Extracellular
**1-Methoxypropan-2-yl acetate** γ	6.47	58/72	850	863	911	108-65-6	NA	NA
**1,4-Xylene** γ	6.57	91	865	867	907	106-42-3	HMDB59924	Membrane
**Cyclohexanone** S *****	7.20	98/70	894	891	904	108-94-1	HMDB03315	NA
**2-Methylheptan-2-ol***	7.25	59	885	893	748	625-25-2	NA	NA
**Unknown 1** γ	8.30	58/69	NA	930	NA	NA	NA	NA
**4-Methylheptan-2-one ***	8.44	58/59	943	934	928	6137-06-0	NA	NA
**Unknown 2** γ	9.22	59	NA	961	NA	NA	NA	NA
**5-Methylheptan-2-one***	9.35	58/71	971	965	714	18217-12-4	NA	NA
**Unknown 3** γ	9.95	58/85	NA	985	NA	NA	NA	NA
**Hexanoic acid** S*	10.00	87	990	987	959	142-62-1	HMDB00535	Cytoplasm<br>Extracellular<br>Membrane
**2-Methylpentan-1,3-diol ***	10.65	57/89	1005	1009	682	149-31-5	NA	NA
**Unknown 4** γ	10.87	56	NA	1016	NA	NA	NA	NA
**Phenylmethanol ***	11.33	77	1036	1031	799	100-51-6	HMDB03119	NA
**4,6-Dimethylheptan-2-one** γ	11.90	78/84	1045	1050	836	19549-80-5	NA	NA
**2,4-Dimethylheptan-1-ol***	11.91	57	1030	1050	731	18450-73-2	NA	NA
**Phenylethanol***	12.14	77/122	1055	1058	847	1517-69-7	HMDB32619	Cytoplasm<br>Extracellular
**2,6-Dimethyloct-7-en-2-ol** γ	12.50	67	1064	1070	851	18479-58-8	NA	NA
**4-Methyl-benzaldehyde***	12.82	91/119/120	1095	1080	953	104-87-0	HMDB29638	Cytoplasm<br>Extracellular

**Chemical name (IUPAC) or common name**	**RT**	**Characteristic ions**	**KI from literature**	**Experimental KI**	**MS-R match**	**Cas number**	**HMDB number**	**Cellular locations**
**1-(3,5-Dimethylfuran-2-yl) ethanone ***	12.99	123/138	1057	1086	760	22940-86-9	HMDB32159	Cytoplasm<br>Extracellular
**Nonan-2-one** [γ]	13.06	58	1092	1088	900	821-55-6	HMDB31266	Membrane
**Methyl benzoate***	13.15	77/105/136	1094	1091	921	93-58-3	HMDB33968	Cytoplasm<br>Extracellular
**Unknown 5** [γ]	14.21	55	NA	1127	NA	NA	NA	NA
**2,7-Dimethyloctan-1-ol (dihydrocitronellol)** [γ]	14.26	56/69	1130	1128	727	15250-22-3	NA	NA
**Unknown 6** [γ]	14.62	55	NA	1141	NA	NA	NA	NA
**Benzyl acetate*** [γ]	15.15	90/135	1164	1159	770	140-11-4	HMDB31310	Cytoplasm<br>Extracellular
**Benzoic acid***	15.33	122	1170	1165	837	65-85-0	HMDB01870	Cytoplasm<br>Extracellular<br>Endoplasmic reticulum
**5-Methyl-2-propan-2-ylcyclohexan-1-ol (DL-menthol)***	15.66	71/75/81	1175	1176	914	2216-51-5	HMDB03352	Extracellular<br>Membrane
**Naphthalene** [γ]	15.83	51/102/127	1182	1182	914	91-20-3	HMDB29751	Membrane
**2-(1-4-Methylcyclohex-3-en-1-yl)propan-2-ol (α-Terpineol)** [γ]	16.14	93/136	1190	1192	869	10482-56-1	HMDB37171	Extracellular<br>Membrane
**6-Ethyl-2-methyldecane** [γ]	16.32	57/71/85	1185	1198	878	62108-21-8	NA	NA
**Decanal** [γ]	16.44	56/57/70	1206	1203	880	112-31-2	HMDB11623	Membrane
**3,7-Dimethyloct-7-en-1-ol (α-citronellol)** [γ]	16.71	67/81	1214	1212	791	6812-78-8	HMDB37171	Extracellular<br>Membrane
**1,3-Benzothiazole** [γ]	16.98	135/108	1229	1222	896	95-16-9	HMDB32930	Cytoplasm<br>Extracellular
**Unknown 7** [γ]	17.03	69/67	NA	1224	NA	NA	NA	NA
**Methyl nonanoate***	17.09	87/129	1225	1226	745	1731-84-6	HMDB31264	Extracellular<br>Membrane
**4-Methyloctanoic acid ***	17.22	99	1208	1231	882	54947-74-9	HMDB34849	Extracellular<br>Membrane
**Ethyl 2-phenylacetate ***	17.44	164	1246	1238	918	101-97-3	HMDB32618	Cytoplasm<br>Extracellular
**Unknown 16 ***	17.52	96	NA	1241	NA	NA	NA	NA
**Unknown 17 ***	17.71	81	NA	1248	NA	NA	NA	NA
**5-Butyloxolan-2-one (γ-octalactone) ***	17.79	85	1261	1251	866	104-50-7	HMDB35422	Cytoplasm<br>Extracellular
**Decan-1-ol** [S γ]	18.30	55/69	1257	1269	888	112-30-1	HMDB11624	Extracellular<br>Membrane

Chemical name (IUPAC) or common name	RT	Characteristic ions	KI from literature	Experimental KI	MS-R match	Cas number	HMDB number	Cellular locations
**Nonanoic acid ***	18.33	73/115	1273	1279	909	112-05-0	HMDB00847	Extracellular Membrane
**Unknown 18 ***	18.55	66/117	NA	1278	NA	NA	NA	NA
**2-Methylundecanal** γ	18.86	58/71	1306	1289	810	110-41-8	HMDB31734	Membrane
**Unknown 8** γ	19.51	140/125/57	NA	1313	NA	NA	NA	NA
**4-Methylnonanoic acid ***	19.50	60/113	1308	1313	798	45019-28	HMDB34849	Extracellular Membrane
**Unknown 9** γ	20.18	72	NA	1338	NA	NA	NA	NA
**2-Methylpropyl 3-hydroxy-2,2,4-trimethylpentanoate** γ	20.33	56/71	1331	1344	783	NA	NA	NA
**5-Pentyloxolan-2-one (γ-Nonanoic lactone) *** γ	20.63	85	1363	1355	890	104-61-0	NA	NA
**Decanoic acid ***	20.94	60/73/129	1373	1367	931	334-48-5	HMDB00511	Extracellular Membrane
**Unknown 19 ***	22.20	73	NA	1416	NA	NA	NA	NA
**Unknown 10** γ	23.12	158	NA	1452	NA	NA	NA	NA
**5-Hexyloxolan-2-one (γ-decalactone) ***	23.32	85	1470	1460	909	706-14-9	NA	NA
**Unknown 11** γ	23.34	85	NA	1461	NA	NA	NA	NA
**Undec-2-enoic acid ***	23.83	99	1479	1481	635	15790-94-0	NA	NA
**6-Pentyloxan-2-one (δ-decalactone) ***	23.97	99	1496	1486	863	705-86-2	HMDB37116	Membrane
**Unknown 12** γ	23.98	57/69	NA	1486	NA	NA	NA	NA
**Unknown 13** γ	24.10	58/119	NA	1491	NA	NA	NA	NA
**Hexadecane** γ	26.60	57/85	1600	1596	907	544-76-3	HMDB33792	Membrane
**Pentadecan-2-one** S γ	28.78	58/59/71	1698	1689	835	2345-28-0	HMDB31081	Membrane
**Unknown 14** γ	28.87	57/85	NA	1693	NA	NA	NA	NA

KI: Kovat indices; HMDB: Human Metabolome Database; NA: not available; S identified with standards; γ present in pH 7; * present in pH 2;

Table 3: Sensitivity and specificity of the discriminant sets obtain at pH 7 and pH 2

	Sensitivity	Specificity	Sensitivity	Specificity
	pH 7		pH 2	
**22RV1 vs PNT2**	100%	100%	69%	100%
**PC3 vs PNT2**	100%	100%	100%	100%
**DU145 vs PNT2**	95%	100%	95%	100%
**LNCaP vs PNT2**	100%	100%	94%	100%

Table 4: Characteristics of prostate cell lines used in this study

	**PNT2**	**22RV1**	**PC3**	**DU145**	**LNCaP**
**Organism**	*Homo sapiens*	*Homo sapiens*	*Homo sapiens*	*Homo sapiens*	*Homo sapiens*
**Age**	33 years	NA	62 years	69 years	50 years
**Ethnicity**	NA	NA	Caucasian	Caucasian	Caucasian
**Tissue**	Prostate	Prostate	Prostate; derived from metastatic site: bone	Prostate; derived from metastatic site: brain	Prostate; derived from metastatic site: left supraclavicular lymph node
**Morphology**	Epithelial	Epithelial	Epithelial	Epithelial	Epithelial
**Culture Properties**	Adherent	Adherent	Adherent	Adherent	Adherent
**Disease**	Healthy	Carcinoma	Grade IV, adenocarcinoma	Carcinoma	Carcinoma
**Tumorigenic**	No	Yes	Yes	Yes	Yes
**AR expression**	Yes	Yes	No	No	Yes
**Metastatic potential**	—	NA	High	Moderate	Low

NA: Not available; AR: Androgen receptor
